# Data on the identification of protein interactors with the Evening Complex and PCH1 in Arabidopsis using tandem affinity purification and mass spectrometry (TAP–MS)

**DOI:** 10.1016/j.dib.2016.05.014

**Published:** 2016-05-14

**Authors:** He Huang, Sophie Alvarez, Dmitri A. Nusinow

**Affiliations:** Donald Danforth Plant Science Center, 975 North Warson Road, St. Louis, MO 63132, United States

**Keywords:** Protein–protein interactions, *Arabidopsis thaliana*, Plant biology, Circadian rhythms, Photoperiodism, Affinity purification, Mass spectrometry

## Abstract

Tandem affinity purification coupled with mass spectrometry (TAP–MS) analysis is a powerful biochemical approach to identify protein–protein associations. Here we describe two datasets generated by a series of TAP–MS analyses to co-purify proteins associated with either ELF3 or ELF4 of the Evening Complex (EC) (“*Identification of Evening Complex Associated Proteins in Arabidopsis by Affinity Purification and Mass Spectrometry*” (Huang et al., 2016a) [1]) or proteins associated with PCH1, which is a newly identified output of the circadian clock to regulate photoperiodic growth in *Arabidopsis thaliana (“PCH1 integrates circadian and light-signaling pathways to control photoperiod-responsive growth inArabidopsis”* (Huang et al. 2016b) [2]). We used either ELF3, ELF4 or PCH1 fused to a C-terminal tandem affinity tag (6xHis-3xFLAG) as baits and conducted purifications in various genetic mutant backgrounds. These data are discussed in recent publications [1,2], and are deposited at the ProteomeXchange Consortium via the PRIDE partner repository with the dataset identifier PRIDE: PXD002606 (for EC) and PRIDE: PXD003352 (for PCH1).

**Specifications Table**

**Table:** 

Subject area	*Biology*
More specific subject area	*Proteomics, Protein–protein interactions, Arabidopsis thaliana, plant biology, circadian rhythms, photoperiodism, affinity purification, mass spectrometry*
Type of data	*Table, figure*
How data was acquired	*Mass Spectrometry (LTQ-Orbitrap Velos Pro)coupled with HPLC* (*U3000 RSLCnano HPLC)*
Data format	*Raw, processed*
Experimental factors	*10–12-day-old seedlings of Arabidopsis thaliana expressing bait proteins were grown under diurnal conditions (light: dark=12 h:12 h with constant temperature (22 °C) and sampled at ZT12 (12 h after light is on).*
Experimental features	*FLAG-His tandem purification using magnetic beads coupled with on-beads trypsin digestion. High-resolution mass spectrometer coupled to nanoHPLC for protein identification and quantification.*
Data source location	*Saint Louis, Missouri, USA*
Data accessibility	*Data are within this article and have been deposited to the ProteomeXchange Consortium via the PRIDE partner repository with the dataset identifier PRIDE: PXD002606 at**http://www.ebi.ac.uk/pride/archive/projects/PXD002606**or dataset identifier PRIDE: PXD003352 at**http://www.ebi.ac.uk/pride/archive/projects/PXD003352*

## 1 Value of the data

• An original TAP–MS protocol for efficiently detecting protein–protein associations in the model plant *Arabidopsis thaliana* from 5 g of starting tissue.
• Combining genetics with TAP–MS reveals if the association between the bait and its associated proteins are affected by different genetic backgrounds, which identified key hub proteins that link the bait to different subgroups in the protein–protein interaction network.
• Data was collected at a specific time of the day (light to dark transition) using diurnally entrained whole seedlings and therefore is of broad interest to people working on the circadian or light signaling pathways of Arabidopsis.
• Control TAP–MS using a heterologous protein (GFP) as bait provides a list of nonspecific binding proteins for experiments using the same TAP–MS protocol.

## 2 Data

Diurnally entrained (12 h light/12 h dark cycles) *A. thaliana* seedlings which express a C-terminal 6xHis-3xFLAG tagged bait protein (ELF3, ELF4 or PCH1) were used in TAP–MS analysis. Co-precipitating proteins specifically associated with the bait at zeitgeber time twelve (ZT 12) were identified and were compared to those found in the control precipitations using tagged GFP as the bait. This data also includes TAP–MS analyses using the same bait proteins in various null mutant backgrounds (*elf3, elf4* or *phyB*) in comparison with the wild type background, in order to reveal the in vivo dependence of ELF3, ELF4 and phyB for specific protein–protein association.

## 3 Experimental design, materials and methods

### 3.1 Plant materials and growth conditions

All plants are in the Columbia (Col) ecotype of *A. thaliana*, carrying a luciferase reporter driven by the promoter of a central clock gene CCA1 (CIRCADIAN CLOCK ASSOCIATED 1). Plants carrying the *CCA1:LUC* reporter are hereafter referred to as wild type. Constructs expressing the bait protein (ELF3, ELF4 or PCH1, fused to 6xHis-3xFLAG tandem affinity tag at the C-terminus) were transformed into wild type plants by floral dipping [3]; homozygous lines with single insertion of the transgene were used for TAP–MS. For ELF3 and ELF4, native promoters were used to express the tagged proteins; the constructs were then transformed into null mutants of ELF3 or ELF4 (*elf3-2* or *elf4-3*, respectively) and successfully complemented the mutant phenotype [1]. For PCH1, the 35S promoter from cauliflower mosaic virus (CaM) was used to overexpress tagged PCH1 in the wild type background and crossed into *elf3-2*, *elf4-2* or *phyB-9* null mutant backgrounds [2]. 12-day-old (for ELF3 and ELF4 TAP–MS) or 10-day-old (for PCH1 TAP–MS) seedlings were grown on a piece of sterilized qualitative filter paper placed on top of 1/2X Murashige and Skoog agar plates (0.8% agar and 3% sucrose, w/v). Light entrainment was set as 12 h light/12 h dark daily cycles, under white light (WL) at 80 µmol m^−2^ s^−1^ and a constant temperature of 22 °C. Whole seedlings were flash frozen in liquid N_2_ at ZT 12, with at least two biological replicates harvested for each sample. A list of all transgenic plants used in this data and numbers of biological replicates for each sample is shown in Table 1.

### 3.2 Sample preparation and protein extraction

**Fig. 1:**
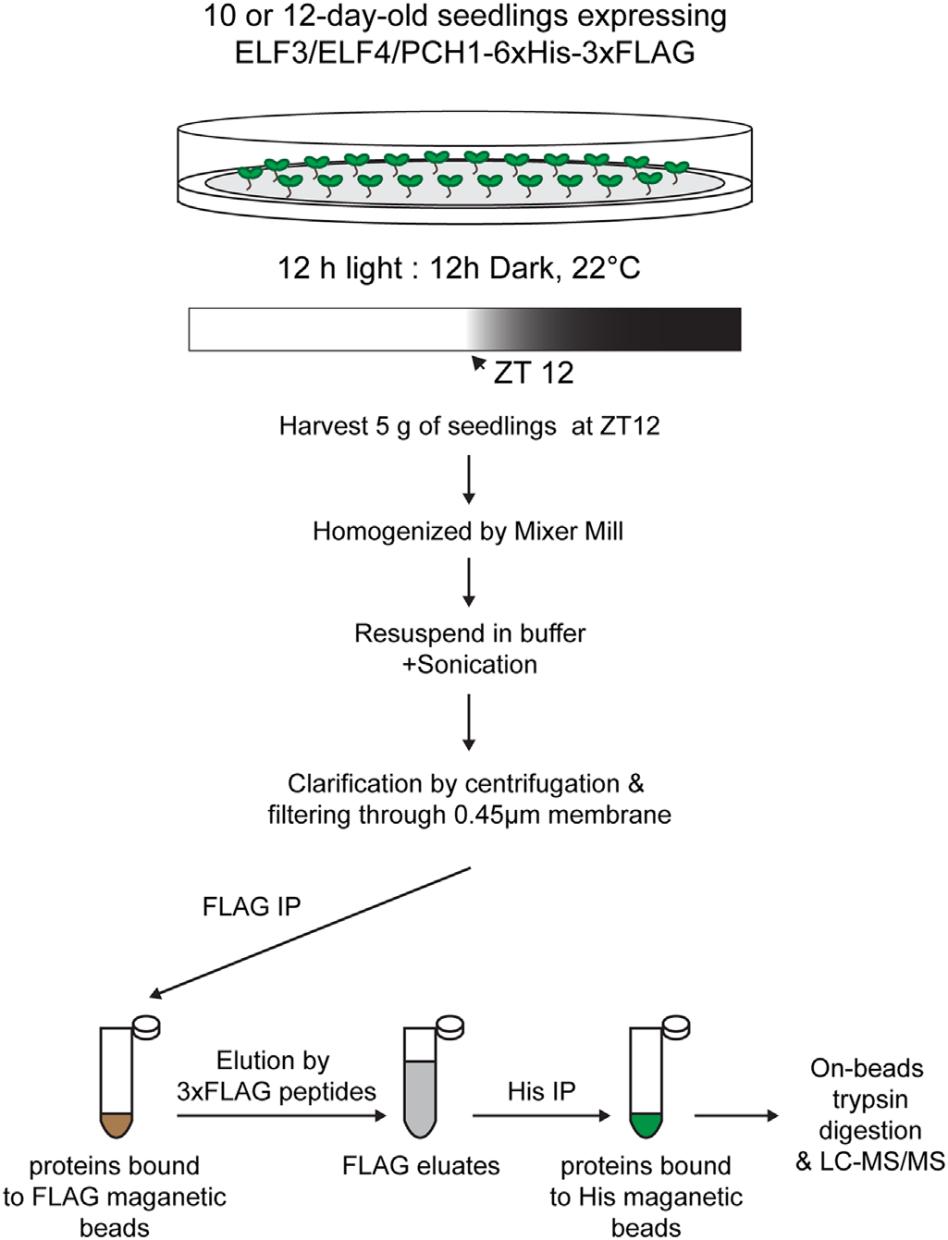
Work-flow of 6xHis-3xFLAG tandem affinity purification.

Tandem affinity purifications using ELF3/ELF4/PCH1-6xHis-3xFLAG as the bait are illustrated in Fig. 1. For each sample, protein extracts were made from 5 g of whole seedlings harvested at ZT 12. Frozen plant tissue was homogenized by a pre-cooled (in liquid N_2_) reciprocal mixer mill (Retsch, Germany) with five rounds of reciprocal shaking (frequency=30 Hz, duration=45 s). Ground tissue of each sample was gently resuspended in 12 mL of SII buffer, which contains 100 mM sodium phosphate, pH 8.0, 150 mM NaCl, 5 mM EDTA, 5 mM EGTA, 0.1% Triton X-100 and supplemented with 1 mM PMSF, 1x protease inhibitor mixture (Roche, Switzerland), 1x Phosphatase Inhibitors II & III (Sigma-Aldrich, St Louis, MO) plus 5 µM MG132 (Peptides International, KY). The fully-resuspended sample was then sonicated twice by a sonicator (Fisher Scientific model FB505, with microtip probe) at 50% power, 1 s on/off cycles for a total duration of 20 s on ice. After two rounds of centrifugation (4 °C at ≥20,000 g for 10 min), clarified supernatants were filtered through a 0.45 µm PVDF membrane (Millex®-HV, MA) to further remove any remaining debris.

### 3.3 FLAG–His tandem affinity purification

Tandem FLAG and His-immunoprecipitations (IP) were carried out to precipitate the bait protein and to co-purify its associated proteins. 42 µg of anti-FLAG antibody (F1804, Sigma-Aldrich) crosslinked to protein G coated magnetic beads (Life Technologies, NY) [4] was incubated with the extract for 60 min (for PCH1) or for 75 min (for ELF3 and ELF4) at 4 °C with rotation (Fig. 1). The beads were then washed twice with 10 mL of SII buffer, transferred to low protein binding 1.5 mL centrifuge tubes, and washed three more times with 900 µL FTH buffer (100 mM sodium phosphate, pH 8.0, 150 mM NaCl, 0.1% Triton X-100). Captured proteins were eluted four times (twice at 4 °C and twice at 30 °C) using 400 µL FTH buffer containing 500 µg/mL 3xFLAG peptide (Sigma-Aldrich, St. Louis, MO). For the following His-IP step, a volume of Talon magnetic beads (70–90 µl, Life Technologies) sufficient to deplete all His tagged bait protein and its associated proteins was incubated with the combined FLAG-IP eluates (~1.6 mL) at 4 °C for 15 min. After binding, Talon resin was washed three times with 900 µL FTH buffer followed by three washes with 900 µL of 25 mM ammonium bicarbonate. After washing, the last bit of liquid from Talon beads was withdrawn; the beads were then stored at −80 °C until digestion and LC-MS/MS analysis.

### 3.4 Protein digestion and liquid chromatography-tandem mass spectrometry (LC-MS/MS)

Proteins binding to the Talon beads were reduced and alkylated using 10 mM TCEP in 50 mM ammonium bicarbonate for 1 h at 37 °C and 20 mM iodoacetamide for 30 min in the dark at RT, respectively. 1 µg of trypsin was added to the beads and samples were incubated at 37 °C overnight. The samples were then acidified and supernatants dried down in new tubes.

The tryptic peptides were dissolved in 5% ACN/0.1% formic acid and 5 µL was injected to an LTQ-Orbitrap Velos Pro (ThermoFisher Scientific, MA) coupled with a U3000 RSLCnano HPLC (ThermoFisher Scientific). The liquid chromatography and mass spectrometer settings used are as previously described [1].

### 3.5 Data analysis

Proteome Discoverer (ThermoFisher Scientific; v.1.4) was used to extract the peaks and generate the mgf files which were submitted to Mascot (Matrix Science, London, UK; v.2.5.0) for database search. The parameters for the search were trypsin for protease, a maximum of two missed cleavages, with a fragment ion mass tolerance of 0.80 Da and a parent ion tolerance of 15 ppm. Deamidation of asparagine and glutamine, oxidation of methionine and carbamidomethyl of cysteine were specified as variable modifications. The databases searched were TAIR10 database (20,101,214 and 35,386 entries) and the cRAP database (http://www.thegpm.org/cRAP/) for PCH1 TAP–MS. For ELF3/ELF4 TAP–MS, database searches were done using the NCBInr database (selected for *A. thaliana*, from September 2014, 228,053 entries) with the other settings as same as in PCH1 TAP–MS.

## Figures and Tables

**Table 1:** Plant materials and number of biological replicates used in this experiment, with reference to the depository raw files.

Genotype	Bio-reps #	RAW files
**ELF3 TAP–MS**		
*elf3-2* [*ELF3pro:ELF3-6xHis-3xFLAG*][*CCA:LUC*]	2	*20130321_120 *min*_DN_ELF3_ELF3; MN_20150427_1*
*elf4-2 elf3-2* [*ELF3pro:ELF3-6xHis-3xFLAG*][*CCA:LUC*]	2	*MN_20150427_5; MN_20150427_6*
*phyB-9 elf3-2* [*ELF3pro:ELF3-6xHis-3xFLAG*][*CCA:LUC*]	2	*20140908_MN_7; MN_20150427_2*
**ELF4 TAP–MS**		
*elf4-3* [*ELF4pro:ELF4-6xHis-3xFLAG*][*CCA:LUC*]	4	*20140908_MN_ELF4_1; 20140908_MN_ELF4_2; 20140908_MN_ELF4_3; 20140908_MN_ELF4_4;*
*elf3-1 elf4-3* [*ELF4pro:ELF4-6xHis-3xFLAG*][*CCA:LUC*]	3	*20140908_MN_3; 20140908_MN_6; 20140908_MN_14*
*phyB-9 elf4-3* [*ELF4pro:ELF4-6xHis-3xFLAG*][*CCA:LUC*]	3	*20140908_MN_1; 20140908_MN_5; 20140908_MN_13*
**PCH1 TAP–MS**		
Col [*35S:PCH1-6xHis-3xFLAG*][*CCA:LUC*]	3	*MN_20130904_Col; 20140908_MN_4; 20140908_MN_16*
*elf3-2* [*35S:PCH1-6xHis-3xFLAG*][*CCA:LUC*]	2	*20131008_MN_elf_2; 20140908_MN_2*
*elf4-2* [*35S:PCH1-6xHis-3xFLAG*][*CCA:LUC*]	2	*MN_20130904_ELF4_2; 20140908_MN_9*
*phyB-9* [*35S:PCH1-6xHis-3xFLAG*][*CCA:LUC*]	2	*20131008_MN_phyB_9; 20140908_MN_10*
**GFP TAP–MS**		
Col [*35S:GFP-6xHis-3xFLAG*][*CCA:LUC*]	4	*20140908_MN_GFP_1; 20140908_MN_GFP_2; 20140908_MN_GFP_4; 20130321_120 *min*_DN_GFP-14*
